# Restrictions and their reporting in systematic reviews of effectiveness: an observational study

**DOI:** 10.1186/s12874-022-01710-w

**Published:** 2022-08-20

**Authors:** Jasmin Helbach, Dawid Pieper, Tim Mathes, Tanja Rombey, Hajo Zeeb, Katharina Allers, Falk Hoffmann

**Affiliations:** 1 Department of Health Services Research, School of Medicine and Health Sciences, Carl Von Ossietzky University Oldenburg, Oldenburg, Germany; 2 Institute for Research in Operative Medicine, Faculty of Health, School of Medicine, Witten/Herdecke University, Cologne, Germany; 3 Faculty of Health Sciences Brandenburg, Brandenburg Medical School Theodor Fontane, Institute for Health Services and Health System Research, Rüdersdorf, Germany; 4 Center for Health Services Research, Brandenburg Medical School Theodor Fontane, Rüdersdorf, Germany; 5 Institute for Medical Statistics, University Medical Centre Göttingen, Göttingen, Germany; 6 Department of Health Care Management, Technische Universität Berlin, Berlin, Germany; 7 Leibniz Institute for Prevention Research and Epidemiology – BIPS, Bremen, Germany; 8 Health Sciences Bremen, University of Bremen, Bremen, Germany

**Keywords:** Reporting quality, Methodological quality, Restriction, Systematic reviews, Effectiveness, Cochrane reviews

## Abstract

**Background:**

Restrictions in systematic reviews (SRs) can lead to bias and may affect conclusions. Therefore, it is important to report whether and which restrictions were used. This study aims to examine the use of restrictions regarding language, publication period, and study type, as well as the transparency of reporting in SRs of effectiveness.

**Methods:**

A retrospective observational study was conducted with a random sample of 535 SRs of effectiveness indexed in PubMed between 2000 and 2019. The use of restrictions and their reporting were analysed using descriptive statistics.

**Results:**

Of the total 535 SRs included, four out of every ten (41.3%) lacked information on at least one of the three restrictions considered (language, publication period, or study type). Overall, 14.6% of SRs did not provide information on restrictions regarding publication period, 19.1% regarding study type, and 18.3% regarding language. Of all included SRs, language was restricted in 46.4%, and in more than half of the SRs with restricted language (130/248), it was unclear whether the restriction was applied during either the search or the screening process, or both. The restrictions were justified for publication period in 22.2% of the respective SRs (33/149), study type in 6.5% (28/433), and language in 3.2% (8/248). Differences in reporting were found between countries as well as between Cochrane and non-Cochrane reviews.

**Conclusions:**

This study suggests that there is a lack of transparency in reporting on restrictions in SRs. Authors as well as editors and reviewers should be encouraged to improve the reporting and justification of restrictions to increase the transparency of SRs.

**Supplementary Information:**

The online version contains supplementary material available at 10.1186/s12874-022-01710-w.

## Background

Systematic reviews (SRs) are becoming increasingly popular in the health sector [1, 2] and are considered the gold standard for summarising the available evidence in clinical research [3]. Traditionally, SRs examine the effectiveness of interventions or therapies [4]. Despite the fact that review methodology has evolved over the years with regard to address a broader range of questions, SRs on effectiveness are still the most common type of SRs [1, 4]. Another development over recent years has been the rapid increase in the number of SRs [1, 2, 5], which may have led to research waste [5, 6]. To avoid this, it is important for published SRs to be of high methodological quality.

Since the aim of SRs is to summarise all available evidence on a topic, it is recommended that they do so regardless of year of publication, study type, language, or other restrictions [7–10]. However, due to lack of time or financial resources, this is not always possible. Language restrictions are particularly frequent [1, 2, 11] and are thus of great relevance and frequently discussed [12, 13]. Furthermore, insufficient language specification in the database and technical limitations regarding language restriction functions in some of the databases can lead to problems when restricting the language during the search process in SRs [7]. In addition, restrictions regarding publication date and study type may increase the potential for systematic bias [7].

Clear reporting is a prerequisite for assessing the methodological quality of SRs and can reduce the risk of bias [14]. Thus, high reporting quality is necessary to identify high quality SRs. To support authors of SRs focusing on randomised controlled trials, the QUOROM statement was published in 1999 [15]. In 2009, it was updated and extended, resulting in the PRISMA statement [16]. Advances in recent years led to a further update of the PRISMA statement in 2020 [17]. Both the original QUOROM and the PRISMA statements explicitly describe the importance of reporting whether SRs apply any restrictions and why. For language restrictions, it is of further importance to know how and where they were used as this can have an impact on search results as well as on reproducibility [7, 12]. Therefore, Pieper and Puljak [12] suggest that the search step in which the language restriction was applied should always be reported, with preference given to restrictions implemented at the full-text screening level. While some literature addresses the transparency of reporting in SRs [1, 2, 5], little research considering restrictions in SRs has been conducted so far.

Therefore, the aim of this research was to examine the use of restrictions regarding language, publication period, and study type as well as transparency of reporting in SRs of effectiveness.

## Methods

This retrospective observational research is based on a previous study [1] for which a protocol is available from the Open Science Framework (https://osf.io/pxjrv/).

In brief, the aim of the previous study was to examine trends in the epidemiology and reporting of published SRs over the past 20 years. SRs indexed between 2000 and 2019 were included. SRs were defined according to the PRISMA-P guideline [18]. To be included, SRs had to provide a clearly stated set of objectives, a systematic search that attempts to identify all studies meeting the eligibility criteria, methods that state the search strategy (or keywords used) and study selection (e.g., eligibility criteria and selection process) as well as a systematic presentation and synthesis of the characteristics and findings of the included studies. Due to limited resources, SRs published in languages other than English were excluded. Using a search strategy from Montori et al. [19], which was adapted according to Moher et al. [20], PubMed was searched on 1 January 2020. The search returned 572,871 results, from which a random sample of 4,000 articles was selected. After titles and abstracts were screened by two independent reviewers (JH, KA, TR, AH), 1,687 articles, one of which was not available, were included in the full-text screening. This led to the exclusion of 555 articles that were not classified as SRs. Finally, 1,132 SRs were included in the original study [1], of which 535 were SRs of effectiveness.

### Data extraction

For the purposes of the current analysis, these 535 SRs on effectiveness were included. Besides the data already extracted in the previous study [1] (journal, publication year, country of corresponding author, number of authors, number of database searched, number of articles included in SRs, meta-analysis performed, number of studies included in the meta-analysis, named article as SR, date of search, assessment of validity, and language restriction), three additional reporting characteristics (reference to PRISMA, full search strategy available, and flow chart available) and six items on the restrictions used (publication period reported, publication period justified, study type reported, study type justified, language restriction justified, and point of language restriction) were considered. Regarding the justification of restrictions, it was recorded which justification the author provided, if any; reasonableness was not assessed in this context. More detailed information on the items can be found in Table 1.

**Table 1:** Operationalisation of the items extracted

Item	Categories	Explanation
Named article as a SR	Yes; No	The authors named their own article as “systematic review” either in the title, abstract, aim or methods section of the article
PRISMA referenced	Yes; No	The PRISMA guideline was explicitly referred to in the abstract or methods section
Date of search reported	Yes; No	The date of search (at least month and year) was reported
Full search strategy available	Yes; No	A complete and reproducible search strategy was available for at least one database. It was not sufficient to present only keywords or search strings. The appendix and/or protocol were only considered if explicitly referred to
Assessment of validity	Yes; No	The validity of the included studies (e.g., risk of bias/methodological quality) was assessed and presented in the SR
Flow chart available	Yes; No	A flow chart was presented in the methods, results or in the appendix (which was only considered if explicitly referenced)
Restriction of publication period	Not reported; Without restriction of period; With restriction of period	Regardless of whether the publication period was restricted, the SR clearly stated which period was considered. If the publication period was restricted, the years under consideration had to be stated. If the publication period was restricted in one database only or the author searched the database from the date of inception (e.g. Medline 1996), the item was classified as “without restriction of period”
Restriction of publication period justified	Yes; No	If the publication period was restricted: In the methods section, an explicit justification why the restriction was chosen had to be provided
Restriction of study type	Not reported; Only RCTs included; NRSI and RCTs included	The included study types were reported
Eligibility of study type justified	Yes; No	Regardless of whether only RCTs or NRSIs & RCTs were considered: In the methods section, an explicit justification why the restriction was chosen had to be provided
Restriction of language	Not reported; Without language restriction; With language restriction	Any language restriction had to be reported in the methods section, either in the eligibility criteria or the search strategy. The appendix and/or protocol were only considered if explicitly referred to
Language restriction justified	Yes; No	If there was a language restriction: In the methods section, an explicit justification why the restriction was chosen had to be provided
Point of language restriction	Unclear; Search strategy; Screening	If there was a language restriction: Was the restriction described in the search strategy or the screening process? If the methodical approach was unclear or it was not possible to retrace whether the language restriction was applied during the search or the screening, the item was evaluated as “unclear”
Failed to report at least one of the restrictions considered	Yes; No	This item is a combination of the three items "restriction of publication period", "restriction of study type", and " restriction of language". If at least one of these items was categorised as “not reported” this item was classified as “Yes”

*SR* Systematic Review, *NRSI* Nonrandomized Studies of Interventions, *RCTs* Randomized Controlled Trials

As the aim of this research was to examine transparent reporting in SRs, authors were not contacted for additional information. Appendices were checked for the search strategy or the flow chart only if they were explicitly referred to in the text.

Data extraction was done by one reviewer (JH). Plausibility checks were performed, and a random sample of 20% of all records was verified by one of the co-authors (DP, TM, TR, KA, and FH).

### Data analysis

Reporting characteristics were analysed and presented using descriptive statistics with a focus on restrictions applied to SRs, including publication period, study type, and language. Frequencies and proportions were calculated for categorical data, and means with standard definitions (SDs) and medians with interquartile ranges (IQR) were calculated for continuous data. Based on the findings from the predecessor study [1], which indicate that the epidemiology and reporting of SRs differ by 5-year period (2000–2004, 2005–2009, 2010–2014, and 2015–2019), country of the corresponding author, and depending on whether the SR was a Cochrane review (defined as a publication in the Cochrane Database of Systematic Review), data were analysed using the same stratifications. Since most of the items considered were explicitly mentioned in the PRISMA checklist [16], which was first published in 2009, a sensitivity analysis was performed for the years 2010–2019. All statistical analyses were conducted with SAS for Windows version 9.4 (SAS Institute INC, Cary, North Carolina).

## Results

### Baseline and reporting characteristics

A total of 535 SRs from 43 different countries were included, with the highest proportion of corresponding authors located in the USA (16.6%), China (16.5%), and the UK (13.5%). On average, 5.2 authors were involved, and 78.9% of the included SRs searched at least three databases. The mean number of included studies was 20.9. Most SRs (65.8%) performed a meta-analysis, of which 95.7% reported the number of included studies, with a mean of 9.0 (Table 2).

**Table 2:** Total frequencies of epidemiological and reporting characteristics in SRs of effectiveness indexed in MEDLINE (via PubMed), 2000–2019

Characteristics ^*^	Total (*n* = 535)
**Journals**	
Cochrane Database Syst Rev	81/535 (15.1%)
PLoS One	14/535 (2.6%)
Medicine (Baltimore)	13/535 (2.4%)
Other	427/535 (79.8%)
**Years**	
2000–2004	25/535 (4.7%)
2005–2009	50/535 (9.4%)
2010–2014	136/535 (25.4%)
2015–2019	324/535 (60.6%)
**Country of corresponding author**	
USA	89/535 (16.6%)
China	88/535 (16.5%)
UK	72/535 (13.5%)
Australia	54/535 (10.1%)
Canada	37/535 (6.9%)
Netherlands	34/535 (6.4%)
Other	161/535 (30.1%)
**Number of authors**	
Mean (SD)	5.2 (2.5)
Median [IQR]	5 [3–7]
**Number of databases searched**	
1	44/535 (8.2%)
2	69/535 (12.9%)
3 +	422/535 (78.9%)
**Included articles (*n* = 534)**	
Mean (SD)	20.8 (26.6)
Median [IQR]	13 [7–25]
**Meta-analysis performed (*n* = 529)**	348/529 (65.8%)
Number of included studies reported (*n* = 333)	333/348 (95.7%)
Mean (SD)	9.0 (8.0)
Median [IQR]	6 [4–11]

^*^ Number of articles included only given when differing from *n* = 535 (e.g., because not reported, not available or not conducted, for instance, in SRs without meta-analysis or empty reviews)

Of all included SRs, 68.8% described themselves as SR in the title, abstract, or methodology, 31.1% referred to the PRISMA guideline, and half of the SRs (55.0%) reported a full search strategy (Table 3). The search date was provided by 89.4% of SRs. An assessment of validity was reported in 77.1%. A flow chart was provided in 77.3% of the SRs.

**Table 3:** Differences in reporting characteristics and the use of restrictions between Cochrane and Non-Cochrane reviews indexed in MEDLINE (via PubMed), 2000–2019

Characteristics ^*^	Cochrane reviews (*n* = 81)	Non-Cochrane reviews (*n* = 454)	Total (*n* = 535)
**Named article as a SR**	19/81 (23.5%)	349/454 (76.9%)	368/535 (68.8%)
**PRISMA referenced**	0/81 (0%)	166/454 (36.6%)	166/535 (31.0%)
**Date of search reported**	79/81 (97.5%)	399/454 (87.9%)	478/535 (89.4%)
**Full search strategy available**	62/81 (76.5%)	225/441 (51.0%)	287/522 (55.0%)
**Assessment of validity**	74/76 (97.4%)	334/453 (73.4%)	408/529 (77.1%)
**Flow chart available**	35/81 (43.2%)	377/452 (83.4%)	412/533 (77.3%)
**Restriction of publication period**			
Not reported	18/81 (22.2%)	60/454 (13.2%)	78/535 (14.6%)
Without restriction of period	55/81 (67.9%)	253/454 (55.7%)	308/535 (57.6%)
With restriction of period	8/81 (9.9%)	141/454 (31.1%)	149/535 (27.9%)
* Restriction justified*	5/8 (62.5%)	28/141 (19.9%)	33/149 (22.2%)
**Restriction of study type**			
Not reported	0/81 (0%)	102/454 (22.5%)	102/535 (19.1%)
Only RCTs included	46/81 (56.8%)	168/454 (37.0%)	214/535 (40.0%)
NRSI and RCTs included	35/81 (43.2%)	184/454 (40.5%)	219/535 (40.9%)
Eligibility of study type justified ^**^	4/81 (4.9%)	24/352 (6.8%)	28/433 (6.5%)
**Restriction of language**			
Not reported	17/81 (21.0%)	81/454 (17.8%)	98/535 (18.3%)
Without language restriction	60/81 (74.1%)	129/454 (28.4%)	189/535 (35.3%)
With language restriction	4/81 (4.9%)	244/454 (53.7%)	248/535 (46.4%)
* Restriction justified*	1/4 (25.0%)	7/244 (2.9%)	8/248 (3.2%)
* Point of language restriction*			
Unclear	1/4 (25.0%)	129/244 (52.9%)	130/248 (52.4%)
Search strategy	0/4 (0%)	51/244 (20.9%)	51/248 (20.6%)
Screening	3/4 (75.0%)	64/244 (26.2%)	67/248 (27.0%)
**Failed to report at least one of the restrictions considered**	29/81 (35.8%)	192/454 (42.3%)	221/535 (41.3%)

Data provided as figures (percent). *SR* Systematic Review, *NRSI* Nonrandomized Studies of Interventions, *RCTs* Randomized Controlled Trials

^*^ The denominator of fractions differs due to missing values (i.e. the information was not available due to a lack of access to the additional material) or as items are not applicable because no studies were included, or no restrictions were applied

^**^ *Study types justified* is based on both categories *Only RCTs included* and *NRSI and RCTs included*

### Restrictions used in SRs

In terms of the restrictions used, 14.6% of the SRs did not specify the publication period, 57.6% included all studies regardless of publication date, and 27.9% applied a restriction (Table 3). Of those SRs restricting their publication periods (149/535), a justification was provided in 22.2% (33/149). The most common reasons for a restricted search period were the attempt to include only recent evidence (27.3%, 9/33), to update a review (21.2%, 7/33), or to reflect the start of scientific relevance (21.2%, 7/33).

Whether restrictions regarding the included study types were applied was not reported in 19.1% of all SRs. In 40.0% of SRs, only randomised controlled trials (RCTs), and in 40.9%, both RCTs and nonrandomized studies of interventions (NRSIs) were included. Where reported (433/535), the selection of study types was justified in 6.5% of SRs (28/433). The main reasons for restrictions were an attempt to include only the highest level of evidence (32.1%, 9/28) or a lack of evidence (32.1% 9/28).

Regarding the language included, 18.3% did not report whether a language restriction was used, 35.3% included all studies regardless of language, and in 46.4%, a restriction was used. Of all SRs that restricted language (248/535), 3.2% provided a justification. All but one SR (87.5%, 7/8) justified language restrictions by limited resources or linguistic capabilities. In one SR (12.5%, 1/8), the restriction was justified by the fact that the main journals in the field are published in English. In 52.4% of all SRs with language restriction (130/248), it was unclear when this step was applied, 20.6% restricted the search strategy, and 27.0% did this during screening. Of all SRs restricting the language in the screening process (67/248), 43.3% (29/67) reported the number of studies excluded due to language, with a mean of 4.6 (SD 5.7, median 2 with a mean of 25.8 included articles, SD 32.3, median 15).

When considering all three restrictions (publication period, study type, or language), 41.3% of all SRs lack information on at least one of them (Table 3).

### Comparisons of cochrane and non-cochrane reviews

When comparing Cochrane (*n* = 81) and non-Cochrane reviews (*n* = 454), differences in reporting and use of restrictions were found (Table 3). Cochrane reviews described themselves less frequently as an SR (23.5% vs. 76.9%), never referred to the PRISMA guideline (0% vs. 36.6%) but were more likely to report the full search strategy (76.5% vs. 51.0%) and search date (97.5% vs 87.9%). Cochrane reviews were less likely to present a flow chart (43.2% vs. 83.4%) but more often provided an assessment of validity (97.4% vs. 73.4%).

Considering the restrictions used, 22.2% of Cochrane reviews and 13.2% of non-Cochrane reviews did not provide any information about the publication period. Overall, 9.9% of Cochrane reviews and 31.1% of non-Cochrane reviews restricted the publication period. While most Cochrane reviews (62.5%, 5/8) justified this restriction, only 19.9% (28/141) of non-Cochrane reviews did so. The type of studies included was reported in all Cochrane reviews versus 77.5% of non-Cochrane reviews. Most Cochrane reviews (56.8%) included only RCTs (37.2% of non-Cochrane reviews).

About every fifth Cochrane review (21.0%) and non-Cochrane review (17.8%) provided no information on whether a language restriction was used. While 4.9% of Cochrane reviews were restricted by language, this proportion was much higher in non-Cochrane reviews (53.7%). In the SRs that were restricted (4/81 Cochrane reviews; 244/454 non-Cochrane reviews), 25.0% of Cochrane reviews and 2.9% of non-Cochrane reviews justified the selected language. More Cochrane than non-Cochrane reviews reported on the step in the research process at which the restriction was used.

Considering all three restrictions, 35.8% (29/81) of Cochrane reviews and 42.3% (192/454) of non-Cochrane reviews lack information on at least one of the restrictions (publication period, study type, or language).

### Differences by country of corresponding author

When stratifying by country of corresponding author, some differences in the reporting of restrictions become apparent (see Additional File 1). Articles with corresponding authors from China restricted the publication period in 15.9% of SRs (14/88), whereas corresponding authors from all other countries restricted the publication period more often, ranging from 26.5% (9/34) in the Netherlands to 38.2% (34/89) in the USA. While SRs from the USA and Australia often did not specify which study type was included (30.3%, 27/89 and 27.8%, 15/54, respectively), 13.6% (12/88) SRs from China lacked information on study type. The highest prevalence of language restrictions was found in SRs from the USA (67.4%, 60/89) and the lowest in SRs from the UK (30.6%, 22/72). Overall, the proportion of articles not reporting at least one of the restrictions considered ranged between 35.2% (19/54) for SRs from Australia to 48.3% (43/89) for those from the USA. A total of 37.5% (33/88) of SRs with a corresponding author from China failed to report at least one restriction (publication period, study type, or language).

### Comparison over time

Looking at the results over time, some trends in the reporting can be identified (Table 4). An increasing number of SRs refer to the PRISMA checklist, named the article as a SR, and presented a flow chart. Some trends are also apparent in the reporting of restrictions. While between 2000 and 2004, one in four SRs (28.0%, 7/25) lack information on the publication period, between 2015 and 2019, only 12.0% (39/324) do so. The proportion of SRs only including RCTs decreased from 60% (15/25) between 2000 and 2004 to 35.5% (115/324) between 2015 and 2019. Regarding language restrictions, on the one hand, the number of SRs reporting no information decreased over time, while the number of SRs reporting a language restriction increased from 24.0% (6/25) between 2000 and 2004 to 52.8% (171/324) between 2015 and 2019. Overall, the number of SRs failing to report at least one of the restrictions considered (publication period, study type, or language) decreased over the years, from 60% (15/25) in 2000–2004 to 40.4% (131/324) in 2015–2019. The proportion of SRs with justifications did not change over the years for any of the three characteristics.

**Table 4:** Reporting characteristics and the use of restrictions in SRs of effectiveness indexed in MEDLINE (via PubMed) by years

Characteristics ^*^	2000–2004 (*n* = 25; 4.7%)	2005–2009 (*n* = 50; 9.35%)	2010–2014 (*n* = 136; 25.4%)	2015–2019 (*n* = 324; 60.5%)
**Named article as a SR**	10/25 (40.0%)	25/50 (50.0%)	89/136 (65.4%)	244/324 (75.3%)
**PRISMA referenced**	0/25 (0%)	0/50 (0%)	15/136 (11.0%)	151/324 (46.6%)
**Date of search reported**	21/25 (84.0%)	46/50 (92.0%)	117/136 (86.0%)	294/324 (90.7%)
**Full search strategy available**	11/25 (44.0%)	23/49 (46.9%)	78/133 (58.7%)	175/315 (55.6%)
**Assessment of validity**	21/25 (84.0%)	30/48 (62.5%)	104/134 (77.6%)	253/322 (78.6%)
**Flow chart available**	0/25 (0%)	16/50 (32.0%)	96/136 (70.6%)	300/322 (93.2%)
**Restriction of publication period**				
Not reported	7/25 (28.0%)	10/50 (20.0%)	22/136 (16.2%)	39/324 (12.0%)
Without restriction of period	14/25 (56.0%)	24/50 (48.0%)	74/136 (54.4%)	196/324 (60.5%)
With restriction of period	4/27 (16.0%)	16/50 (32.0%)	40/136 (29.4%)	89/324 (27.5%)
* Restriction justified*	1/4 (25.0%)	4/16 (25.0%)	10/40 (25.0%)	18/89 (20.2%)
**Restriction of study type**				
Not reported	1/25 (4.0%)	10/50 (20.0%)	19/136 (14.0%)	72/324 (22.2%)
Only RCTs included	15/25 (60.0%)	25/50 (50.0%)	59/136 (43.4%)	115/324 (35.5%)
NRSI and RCTs included	9/25 (36.0%)	15/50 (30.0%)	58/136 (42.7%)	137/324 (42.3%)
Eligibility of study type justified ^**^	1/24 (4.2%)	2/40 (5.0%)	9/117 (7.7%)	16/252 (6.4%)
**Restriction of language**				
Not reported	10/25 (40.0%)	12/50 (24.0%)	22/136 (16.2%)	54/324 (16.7%)
Without language restriction	9/25 (36.0%)	20/50 (40.0%)	61/136 (44.9%)	99/324 (30.6%)
With language restriction	6/25 (24.0%)	18/50 (36.0%)	53/136 (39.0%)	171/324 (52.8%)
* Restriction justified*	1/6 (16.7%)	0/18 (0%)	3/53 (5.7%)	4/171 (2.3%)
* Point of language restriction*				
Unclear	4/6 (66.7%)	10/18 (55.6%)	26/53 (49.1%)	90/171 (52.6%)
Search strategy	1/6 (16.7%)	3/18 (16.7%)	13/53 (24.5%)	34/171 (19.9%)
Screening	1/6 (16.7%)	5/18 (27.8%)	14/53 (26.4%)	47/171 (27.5%)
**Failed to report at least one of the restrictions considered**	15/25 (60.0%)	23/50 (46.0%)	52/136 (38.3%)	131/324 (40.4%)

Data provided as figures (percent). *SR* Systematic Review, *NRSI* Nonrandomized Studies of Interventions, *RCTs* Randomized Controlled Trials

^*^ The denominator of fractions differs due to missing values (i.e. the information was not available due to a lack of access to the additional material) or as items are not applicable because no studies were included, or no restrictions were applied

^**^ *Study types justified* is based on both categories *Only RCTs included* and *NRSI and RCTs included*

The sensitivity analysis for the years 2010 to 2019 supports the trend of improvements in reporting. Overall, 25.0% (13/52) of Cochrane reviews and 41.7% (170/408) of non-Cochrane reviews lack information on at least one of the restrictions (see Additional File 2).

## Discussion

In this study, which analysed a random sample of 535 SRs of effectiveness, we found that 41.3% of all SRs failed to report at least one of the considered restrictions (publication period, study type, or language). Specifically, 14.6% did not report the publication period, 19.1% gave no information about the study type included, and 18.3% did not report the language of studies included. Almost half of the SRs restricted the language of studies included, and for 52.4% of those, it was not clear at which step of the research process the restriction was applied. Only very few SRs provided a justification for the restrictions used. Cochrane reviews more often reported whether restrictions by publication period and study type were done, but in 20% of both Cochrane reviews and non-Cochrane reviews, information on language restrictions was lacking. Furthermore, Cochrane reviews less often presented a flow chart and described their article as SR.

Almost half of all considered SRs restricted the language of studies included. This is a slightly higher proportion than indicated by a range of older studies [2, 5] but quite similar when stratified by year. At first glance, it appears that more SRs restrict their language today than in the past. At the same time, however, reporting has improved over the years, and the proportion of SRs not providing information on language restrictions has decreased. Presumably, rather than reflecting changes in the frequency of applied language restrictions, the observed changes reflect improved reporting. There is conflicting evidence on whether language restrictions increase the risk of language bias [12, 13, 21, 22] and thus might hamper conclusions. Nevertheless, studies show that the likelihood of being published, read, or cited is associated with the language of publication [23–25] and that the influence of language restrictions on the pooled effect estimates can vary between different areas of healthcare research [26, 27]. This complex relationship underlines the need for clear reporting. Pieper and Puljak [12] agreed with the general recommendation [8–10, 16] not to use any language restriction while emphasising that if it is necessary for a good reason (e.g., time or financial resources), the restriction should be applied in the screening process rather than, e.g., during the search. Doing so not only increases transparency but also gives an impression of the relevance of non-English language studies in a research area. Furthermore, it enables other researchers to take up and expand on the study results [7, 12]. However, half of SRs restricting the language did not clearly report at which step of the research process the restriction was applied, and only 27.0% explicitly stated that they restricted the language in the screening process. Where reported, about 5 full text articles were excluded due to language, indicating that non-English language studies might be of importance. For transparency, it is important to not only justify the language restriction, but also to provide the number of excluded studies.

As our results suggest, the reporting of language restrictions needs further improvement. While, the QUOROM statement from 1999 [15] and the PRISMA guideline from 2009 [16] both recommend to report language restrictions [28], in PRISMA 2020 [17], restrictions can only be found in the expanded checklist. This document might be used less frequently because only the core PRISMA 2020 checklist has been translated into other languages, is available as a fillable word document and might be a requirement for submission by journals. Authors should also specify at which step of the research process the language was restricted and how many articles were excluded based on language, as this is not yet included in PRISMA.

While PRISMA 2009 [16] additionally recommended presenting both a flow chart and the full search strategy, we found that 77.1% of included SRs provided a flow chart, and only 55.0% presented a full search strategy for at least one database. It should be noted that PRISMA was introduced in 2009 [16], but the recommendation to show a flow chart was already given in the QUOROM statement from 1999 [15]. On the one hand, most of the reporting-related aspects we assessed were recommended during the whole study period. On the other hand, we found some trends over time, especially for the presentation of a flow chart after PRISMA was introduced. Interestingly, 36.1% of SRs included between 2010 and 2019 referred to the PRISMA statement, while a closer look at the SRs included between 2000 and 2009 reveals that hardly any of them referred to the QUOROM statement. This is supported by previous research indicating that journals have more frequently included the PRISMA guideline in their instructions to authors [29, 30] and that author compliance has increased [31, 32].

Furthermore, QUOROM [15] as well as PRISMA 2009 [16] requires that restrictions regarding publication period and study type must also be reported and justified. Nevertheless, almost 15% of SRs did not report the publication period, and few of them justified the restriction used. As the SRs included in our study showed a high degree of heterogeneity in terms of where and how they stated the publication period, it was not always possible to identify whether a restriction was applied. In addition, the date of intercept for one database varied between SRs. Looking at the type of studies included in SRs, it is notable that most SRs included only RCTs, which are generally considered to be of higher internal validity [33, 34]. However, we found that over the years, the diversity of study types included in SRs has increased, i.e. that fewer SRs include only RCTs. Some studies suggest that NRSIs may be of increasing importance for some areas of research [35, 36].

In accordance with other studies [2, 37–39], we found that aspects considered determinants of methodological quality were more frequently fulfilled by Cochrane reviews than by non-Cochrane reviews. While almost every Cochrane review reported the date of search (97.5%) and provided an assessment of validity (97.4%), only 87.9% and 73.4%, respectively, of non-Cochrane reviews did so. In addition, a full search strategy is available in three quarters of all Cochrane reviews compared to half of non-Cochrane reviews. This is in line with the strict requirements of the Cochrane Handbook [10] and the Methodological Expectations of Cochrane Intervention Reviews (MECIR) [40]. However, MECIR is strongly oriented towards the PRISMA guidelines [16] and is consistent with it in many aspects. While the Cochrane Handbook [10] encourages authors to illustrate the flow of studies through the selection process according to PRISMA [16], less than half of all Cochrane reviews (vs. 83.4%) present a flow chart. This proportion however increased after 2010 for Cochrane reviews (see Additional File 2). Furthermore, it was striking that in some Cochrane reviews (12.3%), the search strategy was not stated and instead, reference was made to a Cochrane search group or a Cochrane register. This approach complicates reproducibility and is inconsistent with the Cochrane Handbook, which requires the presentation of a full search strategy for at least one database. Some other studies indicate that in Cochrane reviews, the authors’ approach deviates from the recommendations of the Cochrane Handbook [41, 42]. Overall, however, few studies are available which compare the methodological approach and reporting of Cochrane reviews versus non-Cochrane reviews. This is important because peculiarities of Cochrane reviews might lead to deficient retrieval [38, 43].

### Strengths and limitations

To our knowledge, few studies focusing on restrictions in SRs exist. General strengths and limitations of the methodological approach have been described in previous research [1]. Including only SRs of effectiveness on the one hand increased homogeneity of the sample. On the other hand, it was sometimes difficult to clearly distinguish these SRs from other types of reviews, which may have led to misclassification. Also, due to limited resources, only English-language articles were included, which may have had an impact on the use of restrictions, especially regarding language. Data were extracted by one reviewer only. However, 20% of the extracted data were checked by a second independent reviewer to ensure methodological quality and to reduce the risk of bias. Additionally, plausibility checks were executed. Despite various efforts, a clear assignment of the restrictions used was difficult for some SRs. When extracting the type of studies included in SRs, a lack of consistency was found regarding the operationalisation of clinical trials. Further, it was not always possible to clearly determine whether a language restriction was used because the supplementary material (such as the search strategy or flow chart) was not accessible for some articles. In most cases, this was due to a missing or incorrect link, inconsistency between journals (some offer only a free full text, others offer only a free appendix, and some always have both available for free), or the problem that the appendix was not available on interlibrary loan. Although most of the recommendations we assessed were already mentioned in the QUOROM statement from 1999, some were first introduced in 2009 with PRISMA. However, more than 85% of included SRs were published between 2010 and 2019, and we also presented analyses stratified by years (see Table 4 and Additional File 2).

## Conclusion

We found that there is a lack of transparency in the reporting of restrictions in SRs of effectiveness. Four out of ten SRs lack information on at least one of the three restrictions considered (publication period, study type, and language). Overall, 20% do not provide information on language restrictions, and among the remaining SRs, many fail to report the step of the research process at which the restriction was applied. Only a small number of SRs justified the restriction used. Furthermore, we also found weaknesses in the reporting of Cochrane reviews. Authors as well as editors and reviewers of all SRs should be encouraged to improve the reporting of restrictions. Explicitly addressing the reporting of restrictions in the main PRISMA checklist in the future may assist authors in doing so.

## Supplementary Information

**Additional file 1:** **eTable 1. **Reporting characteristics and the use of restrictions in SRs of effectiveness indexed in MEDLINE (via PubMed) by country of corresponding author, 2000-2019

**Additional file 2:** **eTable 2. **Differences in reporting characteristics and the use of restrictions after the publication of PRISMA in 2009 (via PubMed)

## Data Availability

The datasets used and/or analysed during the current study are available from the corresponding author on reasonable request.
