# Supplementary material for: Restrictions and their reporting in systematic reviews of effectiveness: an observational study
Source: BMC Med Res Methodol. 2022 Aug 20;22:230. doi: 10.1186/s12874-022-01710-w (PMC9392276; doi:10.1186/s12874-022-01710-w)
Supplement: Supplementary file 1 — Additional file 1: eTable 1. Reporting characteristics and the use of restrictions in SRs of effectiveness indexed in MEDLINE (via PubMed) by country of corresponding author, 2000-2019 [file 12874_2022_1710_MOESM1_ESM.docx]

**Supplemental Material**

eTable 1. Reporting characteristics and the use of restrictions in SRs of effectiveness indexed in MEDLINE (via PubMed) by country of corresponding author, 2000-2019

| **Characteristics*** | **USA  (n=89)** | **China  (n=88)** | **UK  (n=72)** | **Australia (n=54)** | **Canada (n=37)** | **Netherlands (n=34)** | **Other  (n=161)** |
| --- | --- | --- | --- | --- | --- | --- | --- |
| **Named article as a SR** | 71/89 (79.8%) | 50/88 (56.8%) | 50/72 (69.4%) | 36/54 (66.7%) | 25/37 (67.6%) | 24/34 (70.6%) | 112/161 (69.6%) |
| **PRISMA referenced** | 27/89 (30.3%) | 36/88 (40.9%) | 18/72 (25.0%) | 11/54 (20.4%) | 12/37 (32.4%) | 5/34 (14.7%) | 57/161 (35.4%) |
| **Date of search reported** | 77/89 (86.5%) | 80/88 (90.9%) | 65/72 (90.3%) | 48/54 (88.9%) | 33/37 (89.2%) | 32/34 (94.1%) | 143/161 (88.8%) |
| **Full search strategy available** | 37/87 (42.5%) | 33/85 (38.8%) | 47/68 (69.1%) | 34/52 (65.4%) | 20/36 (55.6%) | 23/34 (67.7%) | 93/160 (58.1%) |
| **Assessment of validity** | 52/89 (58.4%) | 74/88 (84.1%) | 59/71 (83.1%) | 47/53 (88.7%) | 27/36 (75.0%) | 25/34 (73.5%) | 124/158 (78.5%) |
| **Flow chart available** | 70/89 (78.7%) | 80/88 (90.9%) | 45/71 (63.4%) | 44/54 (81.5%) | 24/37 (64.9%) | 20/33 (60.6%) | 129/161 (80.1%) |
| **Restriction of publication period** |  |  |  |  |  |  |  |
| Not reported | 11/89 (12.4%) | 11/88 (12.5%) | 15/72 (20.8%) | 5/54 (9.3%) | 5/37 (13.5%) | 7/34 (20.6%) | 24/161 (14.9%) |
| Without restriction of period | 44/89 (49.4%) | 63/88 (71.6%) | 35/72 (48.6%) | 33/54 (61.1%) | 22/37 (59.5%) | 18/34 (52.9%) | 93/161 (57.8%) |
| With restriction of period | 34/89 (38.2%) | 14/88 (15.9%) | 22/72 (30.6%) | 16/54 (29.6%) | 10/37 (27.0%) | 9/34 (26.5%) | 44/161 (27.3%) |
| *Restriction justified* | 9/34 (26.5%) | 1/14 (7.1%) | 6/22 (27.3%) | 5/16 (31.3%) | 1/10 (10.0%) | 3/9 (33.3%) | 8/44 (18.2%) |
| **Restriction of study type** |  |  |  |  |  |  |  |
| Not reported | 27/89 (30.3%) | 12/88 (13.6%) | 12/72 (16.7%) | 15/54 (27.8%) | 5/37 (13.5%) | 9/34 (26.5%) | 22/161 (13.7%) |
| Only RCTs included | 27/89 (30.3%) | 47/88 (53.4%) | 31/72 (43.1%) | 13/54 (24.1%) | 15/37 (40.5%) | 13/34 (38.2%) | 68/161 (42.2%) |
| NRSI and RCTs included | 35/89 (39.3%) | 29/88 (33.0%) | 29/72 (40.3%) | 26/54 (48.2%) | 17/37 (46.0%) | 12/34 (35.3%) | 71/161 (44.1%) |
| *Eligibility of study type justified*** | 5/62 (8.1%) | 0/76 (0%) | 4/60 (6.7%) | 8/39 (20.5%) | 2/32 (6.3%) | 2/25 (8.0%) | 7/139 (5.0%) |
| **Restriction of language** |  |  |  |  |  |  |  |
| Not reported | 17/89 (19.1%) | 18/88 (20.5%) | 16/72 (22.2%) | 4/54 (7.4%) | 7/37 (18.9%) | 3/34 (8.8%) | 33/161 (20.5%) |
| Without language restriction | 12/89 (13.5%) | 31/88 (35.2%) | 34/72 (47.2%) | 24/54 (44.4%) | 14/37 (37.8%) | 10/34 (29.4%) | 64/161 (39.8%) |
| With language restriction | 60/89 (67.4%) | 39/88 (44.3%) | 22/72 (30.6%) | 26/54 (48.2%) | 16/37 (43.2%) | 21/34 (61.8%) | 64/161 (39.8%) |
| *Restriction justified* | 3/60 (5.0%) | 1/39 (2.6%) | 2/22 (9.1%) | 1/26 (3.9%) | 0/16 (0%) | 1/21 (4.8%) | 0/64 (0%) |
| *Point of language restriction* |  |  |  |  |  |  |  |
| Unclear | 28/60 (46.7%) | 22/39 (56.4%) | 11/22 (50.0%) | 18/26 (69.2%) | 6/16 (37.5%) | 13/21 (61.9%) | 32/64 (50.0%) |
| Search strategy | 14/60 (23.3%) | 11/39 (28.2%) | 3/22 (13.6%) | 4/26 (15.4%) | 4/16 (25.0%) | 3/21 (14.3%) | 12/64 (18.8%) |
| Screening | 18/60 (30.0%) | 6/39 (15.4%) | 8/22 (36.4%) | 4/26 (15.4%) | 6/16 (37.5%) | 5/21 (23.8%) | 20/64 (31.3%) |
| **Failed to report at least one of the restrictions considered** | 43/89 (48.3%) | 33/88 (37.5%) | 33/72 (45.8%) | 19/54 (35.2%) | 14/37 (37.8%) | 16/34 (47.1%) | 63/161 (39.1%) |

*Data provided as figures (percent).* *NRSI=Nonrandomized Studies of Interventions; RCTs=Randomized Controlled Trials*

* The denominator of fractions differs due to missing values (i.e. the information was not available due to a lack of access to the additional material) or as items are not applicable because no studies were included, or no restrictions were applied.

***Study types justified* is based on both categories *Only RCTs included* and *NRSI and RCTs included*.
