# Supplementary material for: Restrictions and their reporting in systematic reviews of effectiveness: an observational study
Source: BMC Med Res Methodol. 2022 Aug 20;22:230. doi: 10.1186/s12874-022-01710-w (PMC9392276; doi:10.1186/s12874-022-01710-w)
Supplement: Supplementary file 2 — Additional file 2: eTable 2. Differences in reporting characteristics and the use of restrictions after the publication of PRISMA in 2009 (via PubMed) [file 12874_2022_1710_MOESM2_ESM.docx]

eTable 2. Differences in reporting characteristics and the use of restrictions after the publication of PRISMA in 2009 (via PubMed)

| **Characteristics*** | **Cochrane reviews (n=52)** | **Non-Cochrane reviews (n=408)** | **Total (n=460)** |
| --- | --- | --- | --- |
| **Named article as a SR** | 13/52 (25.0%) | 320/408 (78.4%) | 333/460 (72.4%) |
| **PRISMA referenced** | 0/52 (0%) | 166/408 (40.7%) | 166/460 (36.1%) |
| **Date of search reported** | 51/52 (98.1%) | 360/408 (88.2%) | 411/460 (89.4%) |
| **Full search strategy available** | 44/52 (84.6%) | 209/396 (52.8%) | 253/448 (56.8%) |
| **Assessment of validity** | 49/49 (100%) | 308/407 (75.7%) | 357/456 (78.3%) |
| **Flow chart available** | 35/52 (67.3%) | 361/406 (88.9%) | 396/458 (86.5%) |
| **Restriction of publication period** |  |  |  |
| Not reported | 7/52 (13.5%) | 54/408 (13.2%) | 61/460 (13.3%) |
| Without restriction of period | 40/52 (76.9%) | 230/408 (56.4%) | 270/460 (58.7%) |
| With restriction of period | 5/52 (9.6%) | 124/408 (30.4%) | 129/460 (28.0%) |
| *Restriction justified* | 3/5 (60.0%) | 25/124 (20.2%) | 28/129 (21.7%) |
| **Restriction of study type** |  |  |  |
| Not reported | 0/52 (0%) | 91/408 (22.3%) | 91/460 (19.8%) |
| Only RCTs included | 28/52 (53.9%) | 146/408 (35.8%) | 174/460 (37.8%) |
| NRSI and RCTs included | 24/52 (46.2%) | 171/408 (41.9%) | 195/460 (42.4%) |
| *Eligibility of study type justified*** | 4/52 (7.7%) | 21/317 (6.6%) | 25/369 (6.8%) |
| **Restriction of language** |  |  |  |
| Not reported | 7/52 (13.5%) | 69/408 (16.9%) | 76/460 (16.5%) |
| Without language restriction | 44/52 (84.6%) | 116/408 (28.4%) | 160/460 (34.8%) |
| With language restriction | 1/52 (1.9%) | 223/408 (54.7%) | 224/460 (48.7%) |
| *Restriction justified* | 0/1 (0%) | 7/223 (3.1%) | 7/224 (3.1%) |
| *Point of language restriction* |  |  |  |
| Unclear | 0/1 (0%) | 116/223 (52.0%) | 116/224 (51.8%) |
| Search strategy | 0/1 (0%) | 47/223 (21.1%) | 47/224 (21.0%) |
| Screening | 1/1 (100%) | 60/223 (26.9%) | 61/224 (27.2%) |
| **Failed to report at least one of the restrictions considered** | 13/52 (25.0%) | 170/408 (41.7%) | 183/460 (39.8%) |
|  |  |  |  |

*Data provided as figures (percent).* *NRSI=Nonrandomized Studies of Interventions; RCTs=Randomized Controlled Trials*

*The denominator of fractions differs due to missing values (i.e. the information was not available due to a lack of access to the additional material) or as items are not applicable because no studies were included, or no restrictions were applied.

***Study types justified* is based on both categories *Only RCTs included* and *NRSI and RCTs included*.
